# A rare Down syndrome foetus with *de novo* 21q;21q rearrangements causing false negative results in non-invasive prenatal testing: a case report

**DOI:** 10.1186/s12920-020-00751-8

**Published:** 2020-07-06

**Authors:** Hui-Hui Xu, Mei-Zhen Dai, Kai Wang, Yang Zhang, Fei-Yan Pan, Wei-Wu Shi

**Affiliations:** 1 Prenatal Diagnosis Center, Taizhou Hospital, Wenzhou Medical University, Zhejiang, China; 2 Medical Research Center, Taizhou Hospital, Wenzhou Medical University, Zhejiang, China; 3 Department of Gynecology and Obstetrics, Taizhou Hospital, Wenzhou Medical University, Zhejiang, China

**Keywords:** Non-invasive prenatal testing (NIPT), False negative, Down syndrome, 21q;21q rearrangements

## Abstract

**Background:**

Non-invasive prenatal testing (NIPT) has been established as a routine prenatal screening to assess the risk of common foetal aneuploidy disorder (trisomy 21, 18, and 13). NIPT has high sensitivity and high specificity, but false positive and false negative results still exist. False negative NIPT results involving Down syndrome are rare, but have a high clinical impact on families and society.

**Case presentation:**

We described a case of a foetus that tested “negative” for trisomy 21 (*Z*-score was 0.664) by NIPT based on the semiconductor sequencing platform (SSP). The foetal fraction of cell-free DNA was 16.9%; this percentage was much larger than the threshold of 4% for obtaining accurate NIPT results. However, postnatally, the newborn was diagnosed with Down syndrome with the 46,XY,der(21;21)(q10;q10),+ 21 karyotype.

**Conclusions:**

We presented a case of false negative NIPT results, which may occur through biological mechanisms rather than poor quality, technical errors or negligence. It is imperative for clinical geneticists and their patients to understand that NIPT is still a screening test.

## Background

Non-invasive prenatal testing (NIPT) based on massively parallel shotgun sequencing (MPSS) is widely available as a common clinical screening to assess the risk of foetal aneuploidy disorder (trisomy 21, 18, and 13) during pregnancy [1, 2]. NIPT evaluates cell-free foetal DNA (cfDNA) fragments in the maternal circulation, which can be detected as early as 9 weeks of gestation, and the cfDNA fraction in the maternal plasma is approximately 5–20% between 10 and 26 weeks of gestation [3, 4].

A meta-analysis of 117 cohort studies based on NIPT in singleton pregnancies demonstrated sensitivity and specificity for trisomy 21 (T21, Down syndrome) of 99.4 and 99.9%, for trisomy 18 (T18, Edwards syndrome) of 97.7 and 99.9%, and for trisomy 13 (T13, Patau syndrome) of 90.6 and 100%, respectively [5]. NIPT has high sensitivity and high specificity in assessing the risk of common foetal aneuploidies. However, foetal cfDNA in maternal plasma originates from apoptotic placenta cytotrophoblasts [6]. Therefore, NIPT results may not always represent the actual foetal karyotype of all foetuses; false positive and false negative results still exist [7–10]. NIPT is a screening method, and positive results should be confirmed by amniocentesis and karyotyping, which are recommended by the American College of Obstetrics and Gynecology (ACOG) and the Society for Maternal Fetal Medicine (SMFM). The common causes of false positive NIPT results include placental mosaicism [8], foetal chromosome rearrangement, vanishing twin or co-twin demise [11], and familial chromosome abnormalities or malignancy [12].

In contrast, false negative NIPT results involving foetal aneuploidies are rarely found in follow-ups with large numbers of clinical cases [13, 14]. It is generally believed that the main cause of false negatives is the low foetal fraction of cfDNA in the maternal circulation, which is related to higher-weight women, earlier gestational age (< 10 weeks), and prolonged storage of blood samples prior to processing (> 24 h) [15, 16]. A few false negative NIPT results were confirmed as placental mosaicism according to a retrospective audit of a large number of chorionic villus samples (CVS) [8–10, 17]. In addition, a low foetal fraction of cfDNA and placental mosaicism have been implicated in some false negative results, while other instances remain unexplained [18]. There is little information on these factors affecting the false negative NIPT results. Notably, false negatives are more likely to cause clinical misdiagnosis, and it is important to study the causes of false negative results in NIPT. Clinical geneticists should be aware of these false negative situations, and patients should be informed of the possibility of discordant results between NIPT and subsequent cytogenetic analyses.

In this study, we reported a case of a foetus that tested “negative” for trisomy 21 by NIPT but was postnatally diagnosed with Down syndrome with a 46,XY,der(21;21)(q10;q10),+ 21 karyotype via newborn blood.

## Case presentation

The patient was a one-month-old male who visited Taizhou Hospital with his parents. He was born at 37 + 4 weeks gestation by normal vaginal delivery, weighed 2850 g and had a length of 50 cm. Down syndrome was suspected based on the typical physical features seen at birth, including a flat nasal bridge and up slanting palpebral fissures. Peripheral blood karyotyping confirmed the diagnosis of Down syndrome with the 46,XY,der(21;21)(q10;q10),+ 21 karyotype in all clones. Complications observed in the neonatal period included neonatal haemolysis, atrioventricular septal defect (AVSD), and patent ductus arteriosus (PDA).

**Fig. 1:**
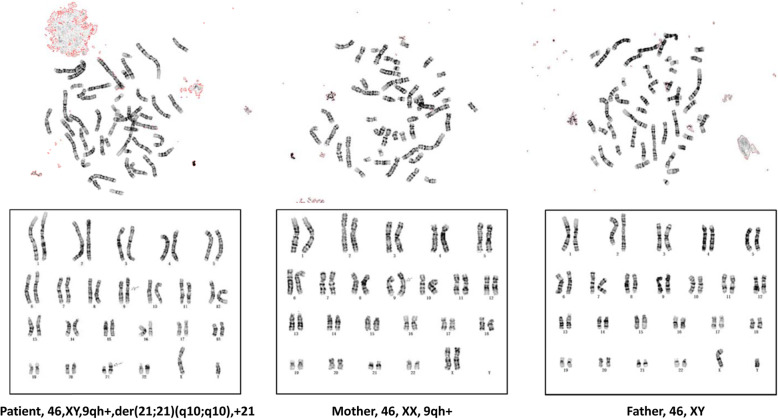
G-banded karyotypes of the patient and his parent

The patient’s mother, who was 30 years old with G3P1A1 (height 163 cm, weight 59.0 kg, BMI 22.2), had a 4-year-old healthy child but suffered one spontaneous abortion. During pregnancy, first-trimester ultrasound examination showed a single gestational sac with a heartbeat, and the foetal nuchal translucency (NT) was normal (1.1 mm) at 12 + 4 weeks gestation. Second-trimester maternal serum screening showed a calculated risk of 1/592 for trisomy 21 at 16 + 3 weeks gestation. NIPT results indicated that the foetus was at “low risk” for each of the three common trisomies (*Z*-score for T21 = 0.664, T18 = 0.424, and T13 = 0.205) at 17 + 5 weeks gestation, and the foetal fraction of cfDNA was 16.9%. The unrelated parents were healthy and had no medical histories. Parental karyotyping showed that the mother was a carrier of the 46,XX karyotype and that the father was a carrier of the 46,XY karyotype (Fig. 1).

## Discussion and conclusions

Trisomy 21, the chromosomal basis of Down syndrome [OMIM #190685], is the most common foetal aneuploidy and accounts for approximately 3% of all prenatal karyotyping [19, 20]. Available data on Down syndrome indicate that 94–96% of cases have standard karyotypes (47, XN, + 21), 2–4% have foetal chromosomal structural rearrangements, and less than 1% have familial chromosome abnormalities or mosaicism [20, 21]. Robertsonian translocation 14q;21q and chromosome 21q;21q rearrangements are the most common abnormalities in foetal chromosomal rearrangement and occur with equal frequencies. More than 95% of 21q;21q rearrangements in Down syndrome arise *de novo* [22]. It is noteworthy that *de novo* 21q;21q rearrangements are overrepresented (28%, 8/29) among false negative NIPT results involving Down syndrome; this percentage is an approximately 14-fold increase over the 2% of live births with Down syndrome [23]. Understanding the biological factors behind this false negative result of *de novo* 21q;21q rearrangements can improve prenatal diagnostic follow-up and genetic counselling.

To explore the possible causes of our false negative case using NIPT, we first analysed the known factors that cause a low foetal fraction of cfDNA. The patient’s mother underwent NIPT at 17 + 5 weeks gestation, with a weight of 59.0 kg and a BMI of 22.2. Second trimester ultrasound examination did not find the presence of a vanishing twin. Plasma separation of the blood sample was completed within 8 h of processing. The foetal fraction of cfDNA was 16.9%; this percentage was much larger than the threshold of 4% for obtaining accurate NIPT results based on the semiconductor sequencing platform (SSP) [24]. Then, we investigated the parental karyotype, which showed that the mother was a carrier of the 46,XX karyotype and that the father was a carrier of the 46,XY karyotype; thus, this case with of 21q;21q rearrangement was a *de novo* foetal chromosomal 21q rearrangement. However, we could not identify this false negative NIPT case due to potential mosaicism, as we did not collect tissue samples from the umbilical cord and placenta for further examination after delivery. Unfortunately, the patient’s mother did not undergo a high-resolution ultrasound examination at 24 weeks gestation.

To our knowledge, this is the 9th report of false negative NIPT results due to chromosome 21q;21q rearrangements. We added this case to the 8 cases that were previously summarized by Huijsdens-van Amsterdam et al. [23] (Table 1). These results showed that false negative NIPT results may occur through biological mechanisms rather than technical limitations or poor quality [7, 14, 23, 25, 26]. 21q;21q rearrangements include isochromosome 21q rearrangements and Robertsonian translocation 21q;21q. Cytogenetic methods cannot distinguish between a true Robertsonian translocation derived from two different homologous chromosomes and an isochromosome composed of genetically identical arms derived from one parental chromosome. Isochromosome 21q arises *de novo* post-fertilization due to centromere mis-division or U-type exchange between sister chromatids. Shaffer et al. [27] found that most 21q;21q rearrangements are isochromosome 21q (88.9%, 32/36), and the remaining rearrangements are true Robertsonian translocations 21q;21q (11.1%, 4/36) accomplished by molecular cytogenetic techniques. Down syndrome due to *de novo* isochromosome 21q is more likely to result in a false negative NIPT result than that due to standard karyotypes (47,XN,+ 21) [23]. A biological cause of the false negative results is almost certainly placental mosaicism arising from the postzygotic formation of 21q;21q rearrangements, which leads to the placental cytotrophoblast having a predominantly normal karyotype [23]. It is important to handle these unexpected false negative NIPT results in prenatal screening.

**Table 1:** Published cases of false negative NIPT results due to chromosome 21q;21q rearrangements

Case number	cfDNA screening technology	Indication for NIPT	Pregnant woman age (yrs)	Blood drawn at GA (wk + d)	Fetal DNA fraction	Z-score for T21	Karyotype	Explanation for false negative NIPT result	Study [reference]
1	MPSS	1/370 risk for T21 by serum screening	32	18 + 0	15.6%	2.04	46,XX,der(21;21)(q10;q10),+ 21	Placental biopsies had 17–53% with T21 mosaicism	Wang et al. (2013) [7]
2	MPSS	Not recorded	26	25	13.4%	Normal	46,XY,der(21;21)(q10;q10),+ 21	Not recorded	Zhang et al. (2015) [14]
3	tMPS	Not recorded	Not recorded	Not recorded	Not recorded	Normal	46,XN,der(21;21)(q10;q10),+ 21	Not recorded	Willems et al. (2016) [24]
4	MPSS	1/140 risk for T21 by serum screening	34	13 + 5	10.5%	0.68	46,XX,i(21)(q10)	Confirmed iso21q; placental trisomy mosaicism confirmed	Oepkes et al. (2016) [25]
5	MPSS	Not recorded	Not recorded	10 + 6	7%	0.63	46,XX,der(21;21)(q10;q10),+ 21	21q rearrangement (presumed iso21q); placenata not available for study	Huijsdens-van Amsterdam et al. (2018) [23]
6	MPSS	1/300–1/700 for T21 by serum screening	Not recorded	12 + 3	3%	0.83	46,XX,i(21)(q10)	Confirmed iso21q; placental not available for study	Huijsdens-van Amsterdam et al. (2018) [23]
7	tMPS	Ultrasound markers	Not recorded	23 + 0	17.2%	Normal	46,XX,der(21;21)(q10;q10),+ 21	21q rearrangement (presumed iso21q); placenata not available for study	Huijsdens-van Amsterdam et al. (2018) [23]
8	tMPS	Ultrasound markers	Not recorded	13 + 0	12.7%	Normal	46,XX,der(21;21)(q10;q10),+ 21	21q rearrangement (presumed iso21q); placenata not available for study	Huijsdens-van Amsterdam et al. (2018) [23]
9	MPSS	1/592 risk for T21 by serum screening	30	17 + 5	16.9%	0.66	46,XY,der(21;21)(q10;q10),+ 21	Placenata not available for study	This study

Notes: cfDNA = cell-free DNA; GA = gestational age; iso21q = isochromosome 21q; MPSS = Massive Parallel Shotgun Sequencing; NIPT = Non-Invasive Prenatal Testing; T21 = Trisomy 21; tMPS = targeted Massive Parallel Sequencing

In conclusion, it is imperative for clinical geneticists and their patients to understand that NIPT is still a screening test. Prior to NIPT, all patients should receive genetic counselling and informed consent on the variety of possible test results, as the risk of false positive or false negative results can occur, to ensure that prenatal patients are able to make more informed decisions regarding the role of NIPT.

## Data Availability

All data generated during this study are included in this published article.

## Abbreviations

AVSD: Atrioventricular septal defect
BMI: Body mass index
CVS: Chorionic villus sampling
DNA: Deoxyribonucleic Acid
MPSS: Massively parallel shotgun sequencing
NIPT: Non-invasive prenatal testing
OMIM: Online Mendelian Inheritance in Man
PDA: Patent ductus arteriosus
